# MFN2 Deficiency Impairs Mitochondrial Transport and Downregulates Motor Protein Expression in Human Spinal Motor Neurons

**DOI:** 10.3389/fnmol.2021.727552

**Published:** 2021-09-16

**Authors:** Yongchao Mou, Joshua Dein, Zhenyu Chen, Mrunali Jagdale, Xue-Jun Li

**Affiliations:** ^1^Department of Biomedical Sciences, University of Illinois College of Medicine Rockford, Rockford, IL, United States; ^2^Department of Bioengineering, University of Illinois at Chicago, Chicago, IL, United States; ^3^MD Program, University of Illinois College of Medicine Rockford, Rockford, IL, United States

**Keywords:** human embryonic stem cell, spinal motor neuron, MFN2, CMT2A, mitochondrial transport

## Abstract

Charcot-Marie-Tooth (CMT) disease is one of the most common genetically inherited neurological disorders and CMT type 2A (CMT 2A) is caused by dominant mutations in the mitofusin-2 (*MFN2*) gene. MFN2 is located in the outer mitochondrial membrane and is a mediator of mitochondrial fusion, with an essential role in maintaining normal neuronal functions. Although loss of MFN2 induces axonal neuropathy, the detailed mechanism by which MFN2 deficiency results in axonal degeneration of human spinal motor neurons remains largely unknown. In this study, we generated MFN2-knockdown human embryonic stem cell (hESC) lines using lentivirus expressing MFN2 short hairpin RNA (shRNA). Using these hESC lines, we found that MFN2 loss did not affect spinal motor neuron differentiation from hESCs but resulted in mitochondrial fragmentation and dysfunction as determined by live-cell imaging. Notably, MFN2-knockodwn spinal motor neurons exhibited CMT2A disease-related phenotypes, including extensive perikaryal inclusions of phosphorylated neurofilament heavy chain (pNfH), frequent axonal swellings, and increased pNfH levels in long-term cultures. Importantly, MFN2 deficit impaired anterograde and retrograde mitochondrial transport within axons, and reduced the mRNA and protein levels of kinesin and dynein, indicating the interfered motor protein expression induced by MFN2 deficiency. Our results reveal that MFN2 knockdown induced axonal degeneration of spinal motor neurons and defects in mitochondrial morphology and function. The impaired mitochondrial transport in MFN2-knockdown spinal motor neurons is mediated, at least partially, by the altered motor proteins, providing potential therapeutic targets for rescuing axonal degeneration of spinal motor neurons in CMT2A disease.

## Introduction

Charcot-Marie-Tooth (CMT) disease is the most common genetically inherited group of motor and sensory neurodegenerative disorders, with a global prevalence of one in every 2,500 people (Skre, 1974; Saporta et al., 2011). The pathogenesis and clinical presentation of CMT are extremely variable and differ based on the individual and inciting genetic mutation, but the disease generally worsens over time as the affected spinal motor neurons degenerate, impairing the neuromuscular junction (Bombelli et al., 2014; Sleigh et al., 2014; Spaulding et al., 2016). It is broadly classified into two subgroups, demyelinating (CMT1) and axonal (CMT2) subgroups (Saporta et al., 2011; Morena et al., 2019). In particular, the predominantly autosomal dominant CMT2A type is the most common form of axonal CMT and has been previously studied in clinical cases as well as animal and *in vitro* models. This subtype of CMT has a variable presentation, but has been associated with more severe phenotypes occurring earlier in life and presenting with a neuropathic phenotype characterized by muscular atrophy, areflexia, pes cavus, vibratory sense loss, and neuropathic pain (Zuchner et al., 2004; Reilly et al., 2011; Vielhaber et al., 2013). Numerous additional features may also be present, including optic atrophy, cognitive impairment, vocal cord paresis, and respiratory insufficiency (Stuppia et al., 2015). CMT2A has been most commonly associated with mutations in the *MFN2* gene, which codes for the GTPase dynamin-like protein, MFN2 (Zuchner et al., 2004; Stuppia et al., 2015; Bertholet et al., 2016).

MFN2 is ubiquitously expressed throughout the human body, including the outer mitochondrial membrane and endoplasmic reticulum in human spinal motor neurons (Burte et al., 2015). The exact mechanism underlying how MFN2 dysfunction contributes to CMT2A has not yet been elucidated. MFN2 is vital in mitochondrial functioning and dynamics, including mitochondrial fusion, transport, and mitophagy (Detmer and Chan, 2007; Stuppia et al., 2015). Fusion of mitochondria is an important, continuous cellular process that promotes the appropriate survival or elimination of damaged mitochondria. Along with mitofusin 1 (MFN1), MFN2 regulates the processes of fusion and fission, which are crucial to proper mitochondrial distribution and shape (Chen et al., 2003; Stuppia et al., 2015). Altered mitochondrial fission and fusion can impair mitophagy, the autophagic process of mitochondrial degradation, and mitochondrial transport, leading to cell death (Itoh et al., 2013). Neurons are highly polarized and need proper axonal transport to maintain their normal functions. One study showed the association between an *mfn2* non-sense mutation in Zebrafish and subsequent impairment in mitochondrial retrograde axonal transport on a cellular level in cultured neurons, as well as reduced motor functioning similar to the phenotypic presentation of CMT2A on an organism level (Chapman et al., 2013). Mutated *MFN2* has also been studied in human embryonic kidney (HEK) cells and neurons derived from human induced pluripotent stem cells (iPSCs) from a patient with CMT2A, revealing abnormal cellular clustering of mitochondria and impaired mitochondrial axonal trafficking (Baloh et al., 2007; Saporta et al., 2015; Rizzo et al., 2016). However, the mechanism by which MFN2 deficiency results in axonal transport defects and axonopathy in human spinal motor neurons remains elusive.

Human pluripotent stem cells, including hESCs and iPSCs, have the capacity to generate any cell type in the human body including neuronal subtypes. These cells offer a unique way to study the cellular and biochemical changes underlying the etiology of various neurodegenerative diseases (Chamberlain et al., 2008; Wang et al., 2013; Xu et al., 2016). Human models of CMT2A have been created and studied on a cellular level using iPSCs from individuals with this disease (Saporta et al., 2015). To better elucidate the role of MFN2 in maintaining human motor neuron and mitochondrial function, we utilized the combination of lentiviral infection and RNA interference (RNAi) in hESC-based models to generate MFN2-knockdown hESC cell lines. These cells were then differentiated into human spinal motor neurons to study their associated mitochondrial function, axonal transport, and morphology. Our data reveal that MFN2 knockdown in human spinal motor neurons results in increased mitochondrial fragmentation and impaired function, including significantly altered morphology, reduced mitochondrial membrane potential, recapitulating CMT2A-specific mitochondrial defects. Using this hESC-based model, we found MFN2 deficiency downregulated motor proteins including KIF1A, KIF5A, and DYNC1I1 for anterograde and retrograde mitochondrial transport, which may contribute to the impaired axonal transport and axonal degeneration of human spinal motor neurons.

## Materials and Methods

### Ethics Statement

All experiments involving hESCs were approved by the University of Illinois Embryonic Stem Cell Research Oversight Committee (ESCRO) and Institutional Biosafety Committee (IBC).

### MFN2 Knockdown Efficiency Test in HEK293FT Cells

**FIGURE 1:**
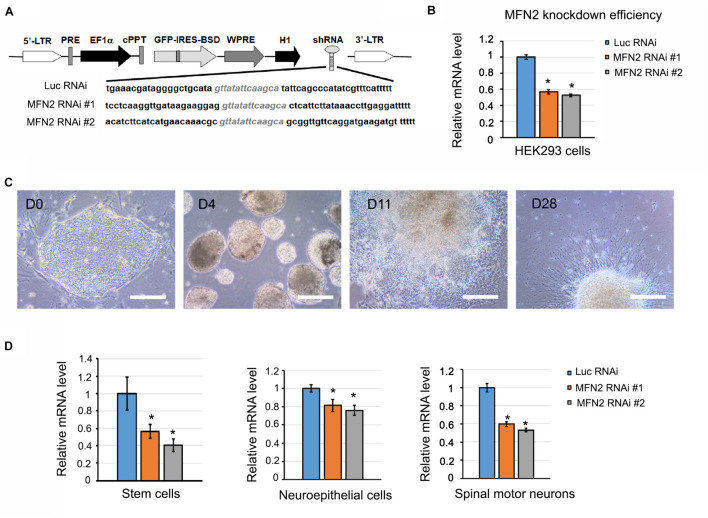
Generation of MFN2 knockdown cell lines. **(A)** Schematic map of pLVTHM containing shRNAs that target *MFN2* and *Luciferase* (Luc, Control). shRNAs that target *MFN2* or *Luc* were inserted into a lentivector pLVTHM under H1 promoter. This vector also expresses GFP-IRES-BSD under EF-1α promoter. GFP is used for visualizing the shRNA-expressing cells, and Blasticidin S deaminase (BSD) is used for drug selection for shRNAs positive hESC clones. 5′ or 3′-LTR: 5′ or 3′- long terminal repeat, PRE: post-transcriptional regulatory element, cPPT for increasing the transduction efficiency: central PPT (polypurine tract), WPRE for increasing transgene expression: WHV (woodchuck hepatitis virus) post-transcriptional regulatory element, H1: H1 promoter, BSD for drug selection: Blasticidin S deaminase. **(B)** *MFN2* knockdown efficiency in HEK293FT cells. **(C)** Differentiation of hESCs to human spinal motor neurons. Cellular stages are indicated at progressive timepoints. D0: hESCs, D4: embryonic bodies, D11: neuroepithelial cells, D28: spinal motor neurons. Scale bar: 50 μm. **(D)** The mRNA expression of *MFN2* in hESCs, neuroepithelial cells, and spinal motor neurons. Data are represented as mean ± SD, *n* = 3. *p < 0.05 compared with Luc RNAi by Dunnett’s test after ANOVA test.

To knockdown MFN2 expression, MFN2 shRNA #1, MFN2 shRNA #2 and Luciferase shRNA (control) were cloned into pLVTHM vectors (Figure 1). The sequence for MFN2 RNAi #1: tcctcaaggttgataagaaggaggttatattcaagcactcattcttataaaccttgaggattttt. MFN2 RNAi #2: acatcttcatcatgaacaaacgcgttatattcaagcagcggttgttcaggatgaagatgtttttt. Luc RNAi (Control): tgaaacgatagg ggctgcatagttatattcaagcatattcagcccatatcgtttcattttt. Before lentivirus transduction was performed in hESCs, knockdown efficiency of MFN2 shRNA was examined in a human embryonic kidney cell line (HEK293FT). This transfection process was carried out using calcium phosphate precipitation. In short, HEK293FT cells plated on 6-well plates were first exposed to a transfection mix of 105 μL ddH_2_O, 15 μL of 2M CaCl_2_, and 120 μL 2XHebs solution. In addition, 3.0 μg MFN2 shRNA or Luciferase shRNA (control) vectors were added. Cells were then incubated in a transfection mix for 6–20 h at 37°C before changing the medium and culturing for 2 days. *MFN2* knockdown efficiency was compared between MFN2 RNAi #1, MFN2 RNAi #2, and Luciferase RNAi (Luc RNAi) using quantitative reverse transcription PCR (qRT-PCR).

### Lentivirus Production and Transduction of hESCs

To generate MFN2 knockdown hESC cell lines, H9 hESCs were infected with pLVTHM lentiviruses (10^6^ transducing units/ml) containing MFN2 shRNA #1 and MFN2 shRNA #2, as well as Luciferase shRNA (as controls). Briefly, to produce high-titer lentivirus, 10 μg of pLVTHM-shRNA lentiviral transfer vector, 7.5 μg of lentiviral vector psPAX2, and 5 μg of pMD2.G (VSV-G envelope protein) were co-transfected into HEK293FT cells (Invitrogen) using the calcium phosphate method (Denton et al., 2018). For transduction with Luciferase and hMFN2 shRNA, H9 hESC pellets were incubated with lentivirus at 37°C for 30 min. The viruses and cell mixture were then transferred to irradiated mouse embryonic fibroblasts (MEF) feeder layers overnight, and the medium was changed the next day. Blasticidin (BSD) was used to select for the transduced GFP-positive hESC clones. MFN2 knockdown efficiency was evaluated by testing mRNA expression using qPCR.

### Spinal Motor Neuron Differentiation From hESCs

Spinal motor neurons were differentiated from hESCs according to our well-established protocol (Li et al., 2005, 2008). In short, MFN2 RNAi #1, MFN2 RNAi #2, and Luc RNAi hESC clones were cultured on MEF feeder layers in hESC media supplemented with 10 ng/mL bFGF. hESCs were dissociated and cultured in suspension in a neural induction medium (NIM) that included DMEM/F12 medium, 1x N2 supplement, and non-essential amino acids with DMH1 (2 μM) and SB431542 (2 μM) for 5 days. During this time, medium was changed every other day. On day 7, the aggregates were attached and cultured in NIM with 0.1 μM of retinoic acid (RA). At day 15–17, neuroepithelial cells were detached and cultured in suspension to form neurospheres in NIM with 1x B27, cAMP (1 μM), RA (0.1 μM), and purmorphamine (0.5 μM). The medium was changed every other day. For terminal differentiation, neurospheres enriched with spinal motor neural progenitor cells were plated on the poly-ornithine and Matrigel-coated coverslips in neurobasal medium (Invitrogen) supplemented with 1x N2, 1x B27, cAMP (1 μM), IGF-1 (10 ng/mL), brain-derived neurotrophic factor (BDNF, 10 ng/mL) and glial-derived neurotrophic factor (GDNF, 10 ng/mL). The medium was changed every other day until the desired day for measurement.

### Western Blotting Assay

Total proteins from D42 spinal motor neurons were extracted using cell lysis buffer containing RIPA lysis buffer (Invitrogen) with 1x Halt Protease Inhibitor Cocktail (Thermo Fisher) and 1 mM phenylmethylsulfonyl fluoride (PMSF). The concentration of total proteins was measured using a Micro BCA Protein Assay Kit (Cat. #: 23235, Thermo Scientific). Total proteins were denatured with 4x Laemmli Sample Buffer (Bio-Rad, Cat. #: 1610747) at 100°C for 10 min. 15 μg total proteins were loaded to 10% SDS-PAGE gel and transferred to a 0.2 μm PVDF membrane at 200 mA for 3 h in a cold environment. Following the blocking with 3% bovine serum albumin (BSA) for 1 h at room temperature, the transferred PVDF membranes were incubated with primary antibodies at 4°C with shaking overnight. The primary antibodies used in this study include mouse IgG anti-MFN2 antibody (Sigma-Aldrich, WH0009927M3,1:500), recombinant rabbit IgG anti-KIF1A antibody (Abcam, ab180153, 1:8,000), rabbit IgG anti-KIF5A antibody (Abcam, ab5628, 1:1,000), rabbit IgG anti-DYNC1I1 antibody (Proteintech, 13808-1-AP, 1:800), and mouse IgG anti-β-ACTIN (Sigma-Aldrich, A5316, 1:10,000). The PVDF membrane was washed with Tris-buffered-saline containing 0.1% tween-20 (TBST) three times and incubated with secondary antibodies donkey anti-mouse or rabbit HRP antibody (Santa Cruz, 1:5,000) for 1 h at room temperature. SuperSignal West Pico Plus chemiluminescent substrate kit (Thermo Scientific, Cat. #: 34579) was used to visualize the bands. The images were obtained by iBright Imaging Systems, and band intensity was quantified and normalized with β-ACTIN using ImageJ software (NIH).

### Immunocytochemistry and Quantification of pNfH Inclusions and Neurite Swellings

Spinal motor neurons were fixed in 4% paraformaldehyde for 20 min and incubated with 0.2% Triton X-100 for 10 min at room temperature. After incubation with 10% donkey serum for 1 h, cells were incubated with primary antibodies at 4°C overnight. Secondary antibodies were used on the second day and incubated at room temperature for 30 min. Hoechst (1 mg/ml) was used to stain the nuclei. Primary antibodies used in this study include rabbit IgG anti-OLIG2 (Abcam, Cat. #: AB109186, 1:1,000), mouse IgG anti-MNR2 (HB9) (Developmental Studies Hybridoma Bank, Cat. #: 81.5 C10, 1:50), mouse IgG anti-ISLET1/2 (Developmental Studies Hybridoma Bank, Cat. #:39.4D5, 1:100), mouse IgG anti-anti-Neurofilament H (NF-H or SMI32, Clone SMI32, Biolegend, Cat. #: SMI-32P, 1:500), rabbit IgG anti-TAU (Sigma, Cat. #: T6402, 1:200), and mouse IgG anti-phosphorylated neurofilament heavy chain (pNfH) (Millipore, Cat. #: MAB1592, 1:1,000). Donkey anti-rabbit Cy3, Donkey anti-mouse Cy3, Donkey anti-mouse Alexa Fluor 647, and Donkey anti-rabbit Alexa Fluor 647 were used as secondary antibodies. To compare between groups, at least three coverslips from each group were used for immunostaining as we described before (Li et al., 2009). At least five fields in each coverslip were imaged using an Olympus confocal microscope or Olympus IX83 microscope.

The pNfH aggregates in human spinal motor neural culture were analyzed following the previous method (Chen et al., 2014). A perikaryal inclusion was defined as an accumulation of immunoreactive staining against pNfH with intensities of threefold higher than that of their local distributions. Neurite swellings were defined as a beading of neurites with positive pNfH that was at least twice the diameter of the neurite. ImageJ (NIH) was used for quantifying these parameters. At least three coverslips from each group were used for immunostaining and at least five fields in each coverslip were imaged using an Olympus IX83 microscope.

### Real-Time Quantitative PCR

D0 hESCs, D11 neuroepithelial cells, and D28 and D42 spinal motor neurons were collected. Total RNA samples were isolated using TRIzol (Invitrogen). 1 μg of RNA was used to generate cDNA using the iScript^TM^ cDNA Synthesis Kit (Bio-Rad). MFN2 mRNA level was analyzed using the Taqman gene expression assay. Mitochondrial transport-associated motor protein gene expression was analyzed by the PowerUp SYBR Green Master Mix (Applied Biosystems) in the QuantStudio 6 Flex Real-Time PCR System (Applied Biosystems). PCR cycling conditions were 50°C for 2 min, 95°C for 3 min, 45 two-step cycles at 95°C for 15 s and 60°C for 60 s, followed by melt-curve stage at 95°C for 15 s, 60°C for 60 s, and 95°C for 15 s. Details of qRT-PCR primers are listed in Supplementary Table 1.

### Mitochondrial Morphological Analysis and Transport by Live-Cell Imaging

To examine the mitochondrial transport, MFN2 RNAi #1, #2, and Luc RNAi spinal motor neurons were plated on poly-ornithine and Matrigel-coated 35 mm dishes (MatTek). D56-old spinal motor neurons were stained with 50 nM MitoTracker Red CMXRos (Invitrogen) for 3 min to visualize mitochondria and then incubated in imaging medium (pH to 7.4) containing 136 mM NaCl, 2.5 mM KCl, 2 mM CaCl_2_, 1.3 mM MgCl_2_, 10 mM HEPES, and 10 mM Glucose. Mitochondrial transport within axons was evaluated through live-cell imaging using an Olympus IX83 microscope equipped with an incubation chamber. Neurons were kept at 37°C with 5% CO_2_ during imaging in the humid chamber. Pictures were captured every 5 s for 5 min. Quantifications were performed using ImageJ with Macros and Multiple Kymograph as described previously (Mou et al., 2020). Motile mitochondria were defined as average velocity ≥ 0.02 μm/s during the entire period (5 min). Mitochondria with an average velocity < 0.02 μm/s were considered stationary (Rumora et al., 2018). The average anterograde and retrograde velocities were analyzed only in the motile mitochondria. Three dishes of spinal motor neurons per group and at least five fields per dish were imaged and analyzed.

The mitochondrial morphology was analyzed by ImageJ (NIH) according to our previously published method (Xu et al., 2016; Mou et al., 2020). In brief, axons were straightened, and the mitochondria were analyzed using the function of Analyze Particles in ImageJ, with mitochondrial area, perimeter, and length being plotted.

### Mitochondrial Membrane Potential

Mitochondrial membrane potential (MMP) was measured based on a previous protocol (Joshi and Bakowska, 2011), and the fluorescent dye tetramethylrhodamine methyl ester (TMRM, Invitrogen) was used because it accumulates in mitochondria based on the MMP (Δψm). Spinal motor neurons were plated on 35 mm glass-bottomed dishes (MatTek). They were washed three times with 5 mM K^+^, 2 mM Ca^2+^ Tyrodes solution, then incubated with 10 nM TMRM in 2 mL Tyrodes solution for 45 min at room temperature in the dark. Live-cell imaging was performed using an Olympus microscope. The fluorescence exposure time and microscope setting were optimized using Luc RNAi neurons, and the same settings were used for all other groups in this study. Five random fields of each dish were tracked and analyzed using ImageJ (NIH). The average pixel intensity of TMRM labeling in each field was measured, followed by background subtraction.

### Phosphorylated Neurofilament Heavy Chain ELISA

The neuron culture medium were collected and concentrated using Amicon Ultra-2 mL Centrifugal Filter Unit (Millipore Sigma). pNfH levels in medium were determined using a commercialized ELISA kit (Cat. #: ELISA-pNF-h-V1, EnCor Biotechnology Inc., United States). In brief, 50 μL of media were added into each well of the ELISA plates and incubated at 4°C overnight. 100 μL of detection antibody solution was added and incubated for 2 h at room temperature, followed by several washes with TBST. The following steps were carried out according to the manufacturer’s instruction and read at 450 nm. pNfH amount in each group was calculated using the standard curve. The pNfH level in the medium was normalized to the total protein of the cultured spinal motor neurons. At least three wells of spinal motor neurons in each group were measured respectively.

### Statistical Analysis

Data were analyzed using Dunnett’s test after ANOVA to determine if the MFN2 RNAi #1 and MFN2 RNAi #2 groups differed significantly from the Luc RNAi control group in this study. Dunnett’s test utilized a significance level of *p* < 0.05.

## Results

### Generation of MFN2-Knockdown Human Embryonic Stem Cell Lines

To gain more insight into the role MFN2 plays in mitochondrial dynamics and human spinal motor neuron degeneration, we established MFN2-knockdown hESC lines to generate spinal motor neurons. This process started with a lentivirus containing shRNA sequences that specifically targeted and knocked down *MFN2* expression. Green fluorescence protein (GFP) in lentiviral vectors served as a marker indicating the positive transduction of lentivirus and consistent expression of *MFN2* shRNA in hESCs. We generated two MFN2-knockdown hESC lines (MFN2 RNAi #1 and #2) using lentivirus infection that expressed shRNA targeting *MFN2* (Figure 1A). The H9 line expressing shRNA targeting luciferase (Luc RNAi), a gene which is not expressed in mammalian cells, was generated as a control. Before lentivirus infection was performed in hESCs, knockdown efficiency of the MFN2-shRNA was examined in HEK cells and MFN2 knockdown efficiency was compared between MFN2 RNAi #1, MFN2 RNAi #2, and Luc RNAi using qRT-PCR. Two shRNA constructs were created and resulted in 44% and 47% reductions in *MFN2* mRNA levels in HEK cells compared to the control, respectively (Figure 1B). We then generated hESC lines expressing Luc RNAi, MFN2 RNAi #1, and MFN2 RNAi #2 using lentiviral transduction. To determine the role of MFN2 in spinal motor neurons, which are specifically affected in CMT2A, MFN2-knockdown hESC lines were differentiated into spinal motor neurons using a well-established method (Li et al., 2005, 2008). Throughout the duration of this differentiation process, four stages were observed at different timepoints. These included stem cell clones at D0, embryonic bodies at D4, neuroepithelial cells with rosettes at D11, and spinal motor neurons at D28 after plating on Matrigel-coated coverslips (Figure 1C). Significantly decreased expression of *MFN2* was observed in three stages of the differentiation process, including stem cells, neuroepithelial cells, and spinal motor neurons (Figure 1D). MFN2 protein levels were determined in spinal motor neurons by western blotting, indicating the significant reduction of MFN2 in MFN2 RNAi #1 and #2 compared with Luc RNAi (Figures 6B,C). These data revealed that the employed lentiviral model was able to effectively knockdown *MFN2* in hESCs and their derivatives, providing a tool for modeling CMT2A disease.

### Effect of MFN2 on Spinal Motor Neuron Differentiation

**FIGURE 2:**
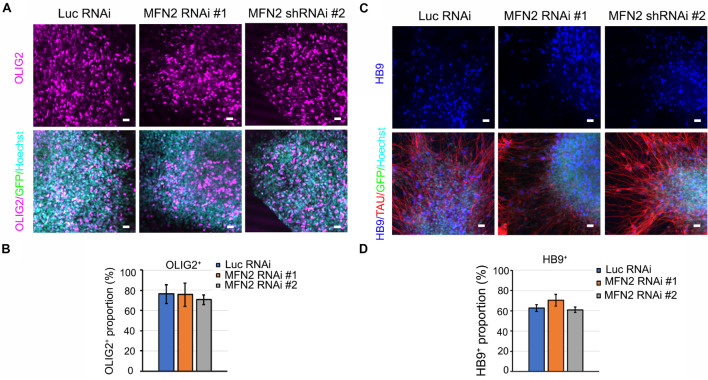
Characterization of motor neurons in Luc RNAi, MFN2 RNAi #1, and MFN2 RNAi #2. **(A)** Immunostaining of spinal motor neuron progenitor markers, OLIG2 (magenta) in D28-old neurons. **(B)** Quantification showing the population of OLIG2^+^ in D28 neural cultures. **(C)** Immunostaining of spinal motor neuron marker, HB9 (blue) and axonal marker TAU (red) in D35-old neurons. Scale bar: 20 μm. **(D)** Quantification of HB9^+^ spinal motor neurons in Luc RNAi, MFN2 RNAi #1, and MFN2 RNAi #2 at D35 of neural culture. Data are represented as mean ± SEM, *n* = 3 independent experiments.

After the effectiveness of our lentivirus model was demonstrated in hESCs, we sought to determine whether reduced *MFN2* expression affects the differentiation process from hESCs to spinal motor neurons. Motor neuron markers for different stages of the differentiation process were measured by immunostaining, including OLIG2 for the spinal motor neuroprogenitor stage and HB9 for the spinal motor neuron stage. At day 28 of the differentiation process, OLIG2 expression in nuclei were observed in about 70% of the cells tested, revealing that most hESCs had differentiated toward the spinal motor neuron lineage and entered the spinal motor neuroprogenitor stage (Figure 2A). GFP in the lentiviral vector indicated the successful expression of shRNA in hESCs (Figure 2A). There was no significant difference between the percentage of cells staining positive for OLIG2 between the MFN2 RNAi #1 and #2 compared with Luc RNAi (Figure 2B). This indicated that MFN2 knockdown may not affect the initial differentiation from hESCs to spinal motor neuron progenitor cells. Further, we determined whether MFN2 knockdown affected the final differentiation into spinal motor neurons by staining the post-mitotic motor neuron marker HB9, and TAU, the axonal marker, at day 35 of the differentiation process (Figure 2C). At this point, about 60% of the spinal motor neurons demonstrated HB9-positivity. There was no significant difference between the percentage of cells staining positive for HB9, indicating that MFN2 knockdown may not affect the differentiation from hESCs to spinal motor neurons (Figure 2D). Furthermore, spinal motor neurons from Luc RNAi and MFN2 RNAi hESC lines were visualized by ISLET1/2 and SMI32, which are additional motor neuron markers (Supplementary Figures 1A,B). These markers did not show obvious alterations after MFN2 knockdown. These data suggest that MFN2 knockdown did not significantly alter the specification and initial differentiation of motor neurons at day 35.

### Altered Mitochondrial Morphology and Function in MFN2-Knockdown Spinal Motor Neurons

**FIGURE 3:**
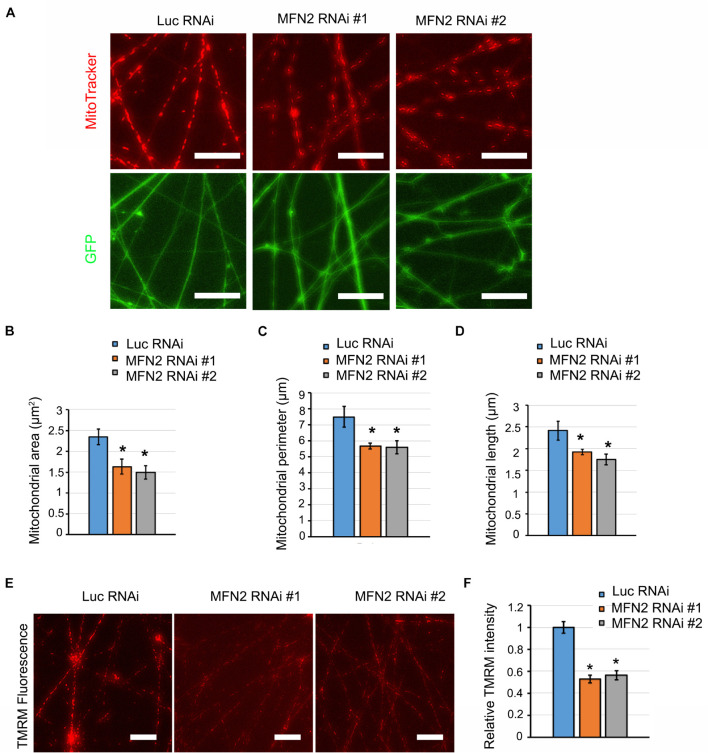
MFN2 knockdown caused defects in mitochondrial morphology and function. **(A)** Mitochondrial visualization in GFP positive neurites using MitoTracker (Red). Scale bar: 20 μm. **(B)** Analysis of mitochondrial area, **(C)** mitochondrial perimeter, and **(D)** mitochondrial length. **(E)** Representative images of TMRM fluorescence in Luc RNAi, MFN2 RNAi #1, and MFN2 RNAi #2 spinal motor neurons. Scale bars: 20 μm. **(F)** Quantification of TMRM average pixel fluorescence intensity. Data are represented as mean ± SEM, *n* = 3 independent experiments. *p < 0.05 compared with Luc RNAi motor neurons.

*MFN2* encoding mitofusin-2, regulates mitochondrial fusion to maintain normal mitochondrial morphology and function (Chen et al., 2003). *MFN2* mutation or loss of function results in CMT2A, leading to mitochondrial impairment and axonal neuropathy of dorsal root ganglion neurons (Misko et al., 2012). To determine whether MFN2 deficit causes mitochondrial impairments in human spinal motor neurons, we analyzed mitochondrial morphology by visualizing mitochondria with MitoTracker (Figure 3A). In MFN2-RNAi neurons, mitochondria appeared to be both smaller and shorter compared with Luc-RNAi neurons. Mitochondrial area, perimeter, and length were significantly decreased in MFN2-RNAi human neurons compared with Luc RNAi neurons (Figures 3B–D), indicating that MFN2 loss led to altered mitochondrial morphology and increased mitochondrial fragmentation. Altered mitochondrial morphology may interfere with mitochondrial functions. We further tested mitochondrial membrane potential (MMP) in MFN2 RNAi spinal motor neurons using TMRM (Figure 3E). A significantly decreased MMP was observed in MFN2 RNAi human spinal motor neurons compared with Luc RNAi group (Figure 3F). These data indicate MFN2 is important for maintaining normal mitochondrial morphology and function, and MFN2 loss induced mitochondrial fragments and led to reduced mitochondrial health.

### MFN2 Knockdown Induced Axonal Degeneration of Spinal Motor Neurons

**FIGURE 4:**
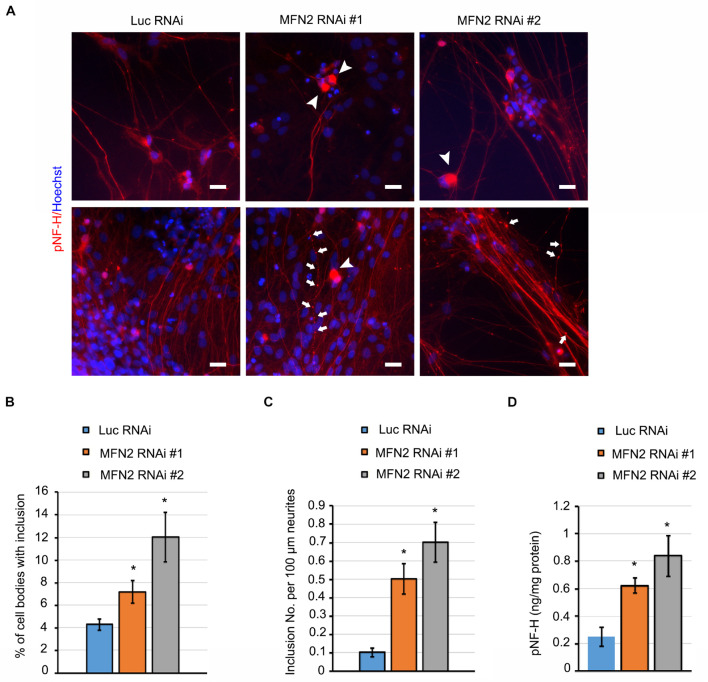
MFN2 deficit induced pNfH inclusion in cell bodies and neurites of spinal motor neurons. **(A)** Immunofluorescent images of pNfH (Red) in spinal motor neurons. Arrowheads indicate pNfH inclusions in the cell bodies; arrow indicates pNF-H inclusions in neurites. Scale bar: 20 μm. **(B)** Quantification of cell bodies with inclusion in MFN2 RNAi #1, MFN2 RNAi #2, and Luc RNAi spinal motor neurons. **(C)** Quantification of inclusion numbers per 100 μm neurites in MFN2 RNAi #1, MFN2 RNAi #2, and Luc RNAi spinal motor neurons. **(D)** ELISA quantification of pNfH level in conditioned cell culture media (normalized to protein). Data are represented as mean ± SEM, *n* = 3 independent experiments. *p < 0.05 compared with Luc RNAi spinal motor neurons.

Mitochondria play a central role in maintaining neural function. Impairment of mitochondria can be a stimulus that leads to axonal degeneration (Court and Coleman, 2012). Neurofilament is a structural component of axonal cytoskeleton in neurons, and hyper-phosphorylation of neurofilament is considered one of the main triggers of its subsequent aggregation in degenerated neurons (Sihag et al., 2007). In some subtypes of CMT disease, neurofilament assembly and transport is linked to degeneration of neurons (Brownlees et al., 2002; Tradewell et al., 2009). pNfH, together with bundled aggregated neurofilament, is a histopathological hallmark for a variety of neurodegenerative diseases (Beck et al., 2012). We have observed that MFN2 deficiency caused mitochondrial defects and dysfunction. We then examined whether MFN2 deficit led to axonal degeneration in spinal motor neurons. In long-term culture of spinal motor neurons, we observed extensive perikaryal inclusions of pNfH in MFN2 RNAi spinal motor neurons (Figure 4A). In addition, frequent scattered, dilated swellings and beadings with the aggregation of pNfH can be clearly visualized in neurites of MFN2 RNAi spinal motor neurons (Figure 4A). The numbers of pNfH inclusions in cell bodies and neurites of MFN2 RNAi spinal motor neurons are significantly increased compared to those in the Luc RNAi group (Figures 4B,C), implicating MFN2 loss in the subsequent axonal degeneration.

In the process of neurodegeneration, axonal remodeling results in the release of a variety of neurofilaments, including pNfH into extracellular fluids, a process which is widely considered as one of the biomarkers of neurodegenerative diseases (Rosengren et al., 1996). In spinal motor neuron culture medium, pNfH level was significantly elevated in MFN2 RNAi group compared with the Luc RNAi group (Figure 4D). These data showed MFN2 loss resulted in the accumulation of pNfH in perikaryon and neurites of spinal motor neurons as well as the increased release of pNfH in the extracellular medium, highlighting that loss of MFN2 contributed to the degeneration of spinal motor neurons.

### MFN2 Deficit Impaired Mitochondrial Transport by Interfering With Motor Protein Expression in Spinal Motor Neurons

**FIGURE 5:**
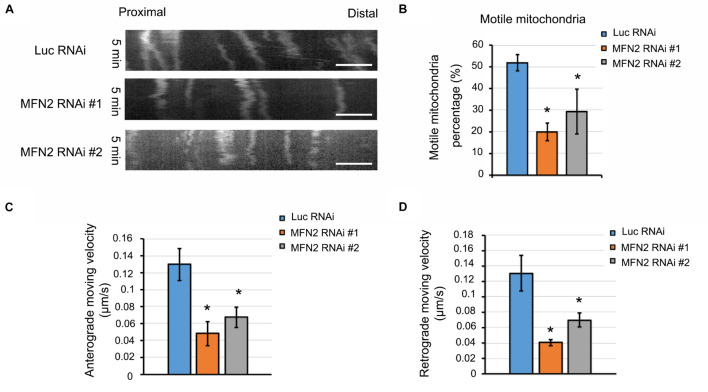
MFN2 knockdown results in defects of mitochondrial transport. **(A)** Representative kymographs of mitochondrial transport in Luc RNAi, MFN2 RNAi #1, and MFN2 RNAi #2 spinal motor neurons. The x-axis indicates axon length from proximal to distal areas. The y-axis indicates time-lapse duration in min (*y* = 5 min). Scale bar: 5 μm. **(B)** Percentage of motile mitochondria in MFN2 knockdown and Luc RNAi spinal motor neurons. **(C)** Average anterograde velocity in MFN2 RNAi #1 and MFN2 RNAi #2 compared with Luc RNAi spinal motor neurons. **(D)** Average retrograde velocity in MFN2 RNAi #1, MFN2 RNAi #2, and Luc RNAi spinal motor neurons. Data are represented as mean ± SEM, over 80 mitochondria from 3 independent experiments were analyzed in each group. *p < 0.05 compared with Luc RNAi.

**FIGURE 6:**
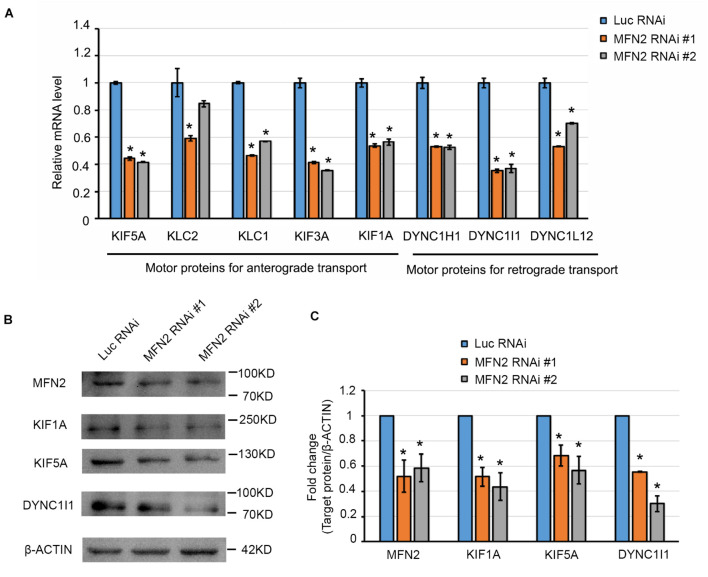
Motor protein expression in MFN2 deficient spinal motor neurons. **(A)** The mRNA expression of anterograde and retrograde transport-associated motor protein genes in MFN2 RNAi #1, MFN2 RNAi #2, and Luc RNAi spinal motor neurons by qRT-PCR. **(B)** MFN2 and important motor proteins expression in D42 spinal motor neurons, including KIF1A, KIF5A, and DYNC1I1. **(C)** Quantification of MFN2, KIF1A, KIF5A, and DYNC1I1 band intensity, β-actin as the reference protein. Data are represented as mean ± SEM, *n* = 3. *p < 0.05 compared with Luc RNAi.

One potential etiology behind the axonal degeneration in CMT disease is the dysregulated mitochondrial positioning due to interrupted transport within axons (Correia et al., 2016). To further elucidate whether MFN2 affects mitochondrial trafficking within axons of spinal motor neurons, we observed mitochondrial transport along axons with live-cell imaging. Kymographs of mitochondrial transport were generated and analyzed by ImageJ (Figure 5A). In the Luc RNAi group, extensive motile mitochondria were clearly observed, and most of the observed mitochondria (70–80% in 5 min) in the MFN2 RNAi neurons remained stationary or barely moving within axons (Figure 5A). Subsequent quantification indicated significant reductions of motile mitochondria in human spinal motor neurons with MFN2 loss compared to controls (Figure 5B). Importantly, both anterograde and retrograde velocity of mitochondria in MFN2-knockdown spinal motor neurons were dramatically reduced, implying MFN2 is necessary for mitochondrial trafficking and its deficit impaired both anterograde and retrograde mitochondrial transport within axons (Figures 5C,D). Motor-based anterograde and retrograde mitochondrial transport along axons is primarily driven by kinesin and dynein, respectively (Pilling et al., 2006). We thus examined the mRNA expression of anterograde and retrograde mitochondrial transport-associated motor proteins in MFN2 RNAi spinal motor neurons (Figure 6A). Interestingly, both kinesin (*KIF5A, KLC1, KIF3A*, and *KIF1A*) and dynein (*DYNC1H1*, *DYNC1I1*, and *DYNC-L12*) gene expression was significantly decreased in human spinal motor neurons with MFN2 loss. The protein levels of KIF1A, KIF5A, and DYNC1I1 expression were significantly decreased in MFN2 RNAi #1 and #2 compared with Luc RNAi spinal motor neurons, suggesting that MFN2 deficiency dramatically reduced axonal transport machinery proteins (Figures 6B,C). Our data suggest MFN2 deficiency in spinal motor neurons impairs mitochondrial transport in both the anterograde and retrograde directions along axons through decreased motor protein expression.

## Discussion

MFN2 deficiency results in CMT2A, a disease that is characterized by axonal neuropathy of peripheral neurons. Using animal models or animal-derived neurons, it has been shown that MFN2 is necessary for mitochondrial fusion and maintenance of normal mitochondrial morphology and distribution (Misko et al., 2012; Bergamin et al., 2016; El Fissi et al., 2018; Rocha et al., 2018). In this study, using a human spinal motor neuron model, we demonstrate that MFN2 loss does not affect the initial differentiation of human spinal motor neurons from pluripotent stem cells, but does result in later mitochondrial fragmentation and dysfunction in these neurons. In addition, MFN2 deficit increases the inclusions of pNfH in cell bodies and neurites of spinal motor neurons, suggesting that MFN2 loss leads to the degeneration of human spinal motor neurons. Importantly, MFN2 deficiency significantly reduces the expression of both kinesin and dynein genes, resulting in decreased anterograde and retrograde mitochondrial motility within axons. Our study demonstrates that loss of MFN2 in human spinal motor neurons leads to axonal degeneration as well as defects in motor-based mitochondrial transport along axons by altering motor proteins expression.

MFN2 plays an essential role in mitochondrial fusion (Chen et al., 2003), endoplasmic reticulum-mitochondria tethering (Filadi et al., 2015; Larrea et al., 2019), and reactive oxygen species (ROS) production (Pich et al., 2005; van Hameren et al., 2019). Proper mitochondrial morphology and positioning allows for local metabolic and energetic needs within axons to be met, which is critical to properly regulate the formation of neuromuscular junctions (Franco et al., 2020). MFN2, located in the mitochondrial outer membrane, regulates mitochondrial fusion to maintain mitochondrial morphology by balancing the process of mitochondrial fission. In vertebrates, both MFN1 and MFN2 are non-redundant and have been shown to be essential in mitochondrial outer membrane fusion, although they are highly homologous and share similar protein domain architecture (Sloat et al., 2019). In our study, MFN2 deficit caused smaller and shorter mitochondria and impaired mitochondrial function, which is consistent with mitochondrial phenotypes observed in CMT2A patient-specific motor neurons (Van Lent et al., 2021). These data confirm that MFN2 plays an essential role in maintaining mitochondrial morphology and function and highlight that its loss leads to altered mitochondrial dynamics and trafficking in human spinal motor neuron axons.

Mutation or loss-of-function of MFN2 causes axonal degeneration and motor deficit in patients (Kijima et al., 2005; Verhoeven et al., 2006). Neurofilament level in biofluid is one of the extremely sensitive biomarkers for many neurological diseases (Khalil et al., 2018). Interestingly, one specific subtypes (CMT2E) is caused by mutations in neurofilament L (Sasaki et al., 2006). Aggregation of pNfH in neurons and its subsequent release into extracellular fluid was increased in MFN2 RNAi neurons, implicating the process of axonal degeneration in our human CMT2A disease models. Although the exact mechanism leading to perikaryal inclusion and aggregation of neurofilament in neurites of MFN2-knockdown spinal motor neurons is not clear, a number of possible explanations have been proposed, including hyperphosphorylation, oxidative stress, or protease activity due to mitochondrial defects (Sihag et al., 2007; Court and Coleman, 2012). The degeneration of spinal motor neurons in our study, as indicated by the aggregation of pNfH in neurites, is consistent with the neurofilament aggregation seen in CMT2A dorsal root ganglion neurons, and abnormal mitochondrial distribution in axons was induced by Mfn2 mutants (Misko et al., 2012). In a transgenic mouse model using the CAMKII promoter to knockout neuronal Mfn2, knockout of Mfn2 in the cortex caused cytoskeleton alterations, including increased MAP2 distribution in cell bodies and hyperphosphorylated Tau in cortical neurons, indicating the importance of Mfn2 in maintaining normal axon structure and function (Jiang et al., 2018). It has been shown that MFN2 loss and mutation induced mitochondria-associated oxidative stress by dysregulating the oxidative phosphorylation (OXPHOS) system in neurons and non-neuron cells (Pich et al., 2005; Jiang et al., 2018; Yao et al., 2019). Mitochondria-linked oxidative stress could modify the post-translation of cytoskeletal proteins and enhance the degradation of microtubule-associated proteins, thereby disturbing normal cytoskeletal networks in neuronal and non-neuronal cells (Kwei et al., 1993; Pang et al., 1996; Akulinin and Dahlstrom, 2003). Pathologically altered mitochondrial distribution may be one of the underlying mechanisms contributing to axonal degeneration in our CMT2A models. Additionally, defective mitochondrial transport could cause the accumulation of mitochondrial fragments in axons, leading to axonal swellings and neuropathy (Sheng and Cai, 2012).

Alterations of other pathways in CMT diseases may contribute to the axonopathy of motor neurons, including post-translational modifications, oxidative stress-activated pathways, and neuroinflammatory responses (Hofer and Wenz, 2014; Fernandez-Lizarbe et al., 2019; Cassereau et al., 2020). Changes to post-translational modifications in CMT diseases have emerged as a critical pathway, including phosphorylation, acetylation, and ubiquitination (Park et al., 2010; d’Ydewalle et al., 2011; Benoy et al., 2018). Targeting these potential pathways or regulating MFN2 activity have showed the possibility of ameliorating CMT disease, including PI3K-Akt signaling pathway, histone deacetylase 6 (HDAC6), and MFN2 agonists (d’Ydewalle et al., 2011; Benoy et al., 2018; Rocha et al., 2018; Van Lent et al., 2021). An imbalanced PI3K-Akt signaling pathway has been found in CMT2A spinal motor neurons and CMT rodent models (Fledrich et al., 2014; Van Lent et al., 2021). A dual leucine kinase (DLK, also known as MAP3K12) inhibitor, GNE-8505 was able to increase the mitochondrial aspect ratio and basal respiration that was reduced in MFN2^R94Q^ spinal motor neurons (Van Lent et al., 2021). HDAC6 is another potential target for rescuing CMT disease. Pharmacological inhibition of HDAC6 rescued axonal transport by increasing alpha-tubulin acetylation and the CMT phenotype of CMT mice with a small heat-shock protein gene (*HSPB1*) mutation (d’Ydewalle et al., 2011). These studies indicate a potentially important role for both post-translational modification and stress-related pathways in CMT diseases.

Given that MFN2 reduction impaired both anterograde and retrograde mitochondrial transport within axons in spinal motor neurons, we further examined how MFN2 loss induced defects in mitochondrial transport. Mfn2 was found to be necessary for mitochondrial transport through its physical interaction with Miro/Milton complexes, which link mitochondria to kinesin motors. In addition, Mfn2 mutant caused slower anterograde and retrograde mitochondrial transport (Misko et al., 2010). The reduced physical interaction between MFN2 and Miro/Milton/kinesin complex could be one of the reasons for interrupted mitochondrial transport in CMT2A spinal motor neurons. Interestingly, our study revealed that the expression of a variety of motor proteins is important for both anterograde and retrograde mitochondrial transport and were significantly reduced in MFN2-knockdown spinal motor neurons. The coordination between kinesin and dynein motors determines the bidirectional trafficking within axons. The reduced kinesin and dynein induced by loss of MFN2 may be another reason for slower mitochondrial trafficking. Although the mechanism of MFN2 deficiency leading to reduced expression of kinesin and dynein-related genes awaits further investigation, our study reveals that MFN2 deficiency in human motor neurons impairs motor protein expression and axonal transport, leading to axonal degeneration and serving as potential therapeutic targets for CMT2A.

## Data Availability Statement

The original contributions presented in the study are included in the article/Supplementary Material, further inquiries can be directed to the corresponding author/s.

## Ethics Statement

All experiments involving hESCs were approved by the University of Illinois Embryonic Stem Cell Research Oversight Committee (ESCRO) and Institutional Biosafety Committee (IBC).

## Author Contributions

X-JL conceived and supervised the study. YM and JD performed MFN2 knockdown stem cell lines generation, stem cell differentiation, mitochondrial transport, and axonal degeneration analysis experiments. ZC performed MFN2 shRNA design and tested gene knockdown efficiency. MJ did the immunostaining and imaging of axonal degeneration. YM, JD, and ZC collected and analyzed data. YM, JD, and X-JL wrote the manuscript with comments from ZC and MJ. All authors read and approved the final manuscript.

## Conflict of Interest

The authors declare that the research was conducted in the absence of any commercial or financial relationships that could be construed as a potential conflict of interest.

## Publisher’s Note

All claims expressed in this article are solely those of the authors and do not necessarily represent those of their affiliated organizations, or those of the publisher, the editors and the reviewers. Any product that may be evaluated in this article, or claim that may be made by its manufacturer, is not guaranteed or endorsed by the publisher.
