# Supplementary material for: MFN2 Deficiency Impairs Mitochondrial Transport and Downregulates Motor Protein Expression in Human Spinal Motor Neurons
Source: Front Mol Neurosci. 2021 Sep 16;14:727552. doi: 10.3389/fnmol.2021.727552 (PMC8482798; doi:10.3389/fnmol.2021.727552)
Supplement: Supplementary file 1 [file Image_1.pdf]

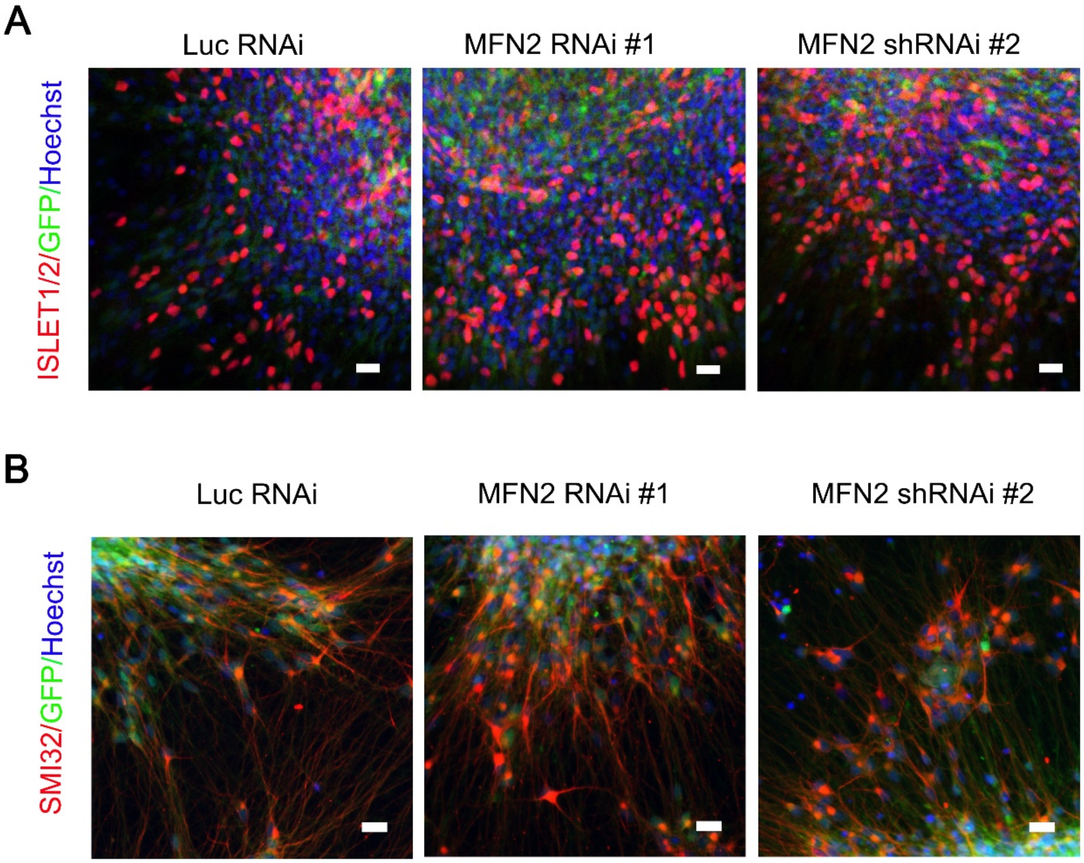

Supplemental Figure 1: Additional motor neuron marker detection in MFN2-knockdown spinal motor neurons. (**A**) ISLET1/2 immunostaining in D35 spinal motor neurons. Red: ISLET1/2, Green: GFP, Blue: Hoechst. Scale bar: 20 μm. (**B**) SMI32 immunostaining in D35 spinal motor neurons. Red: SMI32, green: GFP, blue: Hoechst. Scale bar: 20 μm.
