# Supplementary material for: MFN2 Deficiency Impairs Mitochondrial Transport and Downregulates Motor Protein Expression in Human Spinal Motor Neurons
Source: Front Mol Neurosci. 2021 Sep 16;14:727552. doi: 10.3389/fnmol.2021.727552 (PMC8482798; doi:10.3389/fnmol.2021.727552)
Supplement: Supplementary file 2 [file Table_1.DOCX]

**Supplementary Table 1. List of qRT-PCR primers**

| Gene | Forward Primer | Reverse Primer |
| --- | --- | --- |
| *KIF1A* | GTCCGCCCCTTCAATTCCC | GAGGTGTGCGACCAGTAGG |
| *KIF3A* | GTGTTCGAGCTATTCCTGAACTT | CCTCTAACCTTTGTGTCTGATCC |
| *KIF5A* | ATGTTTTTGACCGTGTATTCCCC | TGAGGGTCGTGCAGCTTTC |
| *KLC1* | GTGAGGCACAGGTTATGATGG | GTTCATCCCGTAGCCACTGAT |
| *KLC2* | AGGGGATGTGTCTGGTCAG | CCTGTGAGGCGTATTGGATCA |
| *KIF1C* | AGGGTTCGGCCCTTTAACG | GTCCCGATACACTTGCTGCT |
| *DYNC1H1* | TTGGGCACTAGGAAATTGATGC | GCAGGGTTGATACGCCACA |
| *DYNC1L12* | GGCTAGTGTTTTACGTGAGCA | TGGGGAACCTTGACAACCTTC |
| *DYNC1I1* | AAAGCTGAGCTAGAGCGCAAA | GTCCTGAACGGGTTCTTTCTTC |
| *GAPDH* | ATGACATCAAGAAGGTGGTG | CATACCAGGAAATGAGCTTG |
